# The epidemiological follow-up process for suspected and confirmed cases of COVID-19 in migrant shelters on the northern border of Mexico from July to December 2020: Between contagion underestimation and containment

**DOI:** 10.3389/fpubh.2022.980808

**Published:** 2023-01-12

**Authors:** María Gudelia Rangel Gómez, Rodolfo Cruz-Piñeiro, Valentina Cappelletti, Ana María López Jaramillo

**Affiliations:** ^1^El Colegio de la Frontera Norte, Tijuana, Mexico; ^2^Comisión de Salud Fronteriza México-Estados Unidos, Tijuana, Mexico

**Keywords:** migrant shelters, northern border of Mexico, COVID-19, epidemiological surveillance, suspected and confirmed COVID-19 cases

## Abstract

**Background:**

Elements associated with an increased risk factor for the contagion of COVID-19 in shelters include the turnover and overcrowding of people, time spent in communal areas, daily supply needs, water availability, and sanitation levels. The “Report on the Effects of the COVID-19 Pandemic on Migrants and Refugees,” shows that factors such as the shortage of food, supplies, water, sanitizing materials, spaces for healthy distancing, financial resources for rent and essential services, and the lack of medical or psychological care complicated providing care for migrants and applicants seeking international protection.

**Objective:**

We describe shelter operations regarding the detection and follow-up of suspected and confirmed COVID-19 cases showing mild symptoms among the migrant population housed in the border cities under study.

**Methods:**

We conducted semi-structured, in-depth interviews with study subjects (people in charge, managers, coordinators, shelter directors) from 22 migrant shelters, and 30 with key informants. We studied the cities of Tijuana (Baja California), Nogales (Sonora), Ciudad Juárez (Chihuahua), Piedras Negras (Coahuila), and Heroica Matamoros (Tamaulipas). The research was based on a qualitative methodological design with an ethnographic approach. The information collected was transcribed and systematized into two tables or analytical templates, one for interviews with study subjects, and another for interviews with key actors.

**Findings:**

Overall, seventy-eight registered shelters provided accommodation services for migrants in the five cities the study focused on: thirty-seven in Tijuana, five in Nogales, twenty-two in Ciudad Juárez, eight in Piedras Negras, and five plus a camp (six in total) in Matamoros. The major concentration of shelters was in Tijuana (47.4%) and Ciudad Juárez (28.2%). At the beginning of the pandemic, only a few shelter facilities met quarantine and isolation guidelines, such as having separate bathrooms and sufficient space to isolate the “asymptomatic” and “confirmed” from close “contacts”. The lack of isolation space and the inability to support the monitoring of patients with COVID-19 posed a challenge for those housed in shelters, forcing many shelters to close or continue operating behind closed doors to avoid becoming a source of infection during the pandemic.

**Discussion and outlook:**

Contrary to speculation, during the onset of the pandemic northern border migrant shelters did not become sources of COVID-19 infection. According to the data analyzed from 78 shelters only seven had confirmed cases, and the classification of “outbreak” was applied only in two facilities. Contagion control or containment was successful as the result of following a preventive containment logic, including the isolation of all suspected but unconfirmed cases, without a clear understanding of the human and financial resources required to maintain isolation areas. However, shelters in the study implemented protocols for epidemiological surveillance, control, and prevention with elements that interfered with monitoring spaces, and processes that caused oversights that resulted in underestimating the number of cases.

**Limitations:**

Due to travel restrictions imposed to prevent and contain coronavirus infections it was impossible to stay on-site in the cities studied, except for Tijuana, or carry-out recordings of migrants' views in shelters.

## 1. Background

On 14 April 2020, the World Health Organization (1) declared that the main purpose of the national and sub-national health systems of every country in the world should be to detect and isolate all suspected cases, trace each contact, and quarantine them to slow down and stop the transmission chains of SARS-CoV-2. Health systems were instructed to conduct robust diagnostic tests and provide adequate and timely care to patients with COVID-19. These objectives were immediately reflected in the standardized guidelines for the detection and epidemiological follow-up of suspected cases of COVID-19 issued by health systems in keeping with the criteria of prevention, control, and epidemiological surveillance.

We analyze how these procedures for epidemiological monitoring and control were followed in contexts of intense population mobility where detection, tracking, isolation, and follow-up faced multiple structural challenges.

The northern border of Mexico is a region with Mexicans and foreigners with various mobility conditions. Recent immigration policies in the United States and Mexico (i.e., border securitization, expedited deportation and expulsion policies, asylum/refugee restrictions), have transformed this region into a transit region, the last great containment filter for national, international, and extra-continental migratory corridors into the United States, and as a waiting territory (2, 3).

This situation was exacerbated in the context of the SARS-CoV-2 pandemic by the establishment of health policies such as Section 265 of U.S. Code Title 42, authorizing U.S. border authorities to expeditiously expel undocumented migrants wishing to enter the United States by land to their last country of transit, rather than their country of origin, even if they had expressed a desire to request asylum (4). In addition, on 21 March 2020, Mexico and the United States agreed to close their shared border to “non-essential” trips, including those involving requests for international protection. All this led to a backlog of cases of people intending to request asylum and asylum seekers in the U.S. under MPP (Migrant Protection Protocols), forced by the Program to wait in Mexican cities on the northern border for their expected U.S. court hearings. By the end of March 2020, more than 52,000 people enrolled in this program, mostly Hondurans, Guatemalans, Cubans, Salvadorans, Venezuelans, and Ecuadorians, were returned to border cities. Also in March 2020, the deportation policy resulted in 19,681 Mexican migrants being forced to return to cities on the northern Mexican border (5).

These expulsion policies produced a humanitarian crisis in Mexican border cities, characterized by high levels of public insecurity and violence (6), by increasing the number of people on the move needing basic assistance.

One of the pillars of the humanitarian system in the region is a network of ~90 shelter and house facilities (7) scattered throughout the main border cities that meet the demand for accommodation services for refugees and migrants.

Before the pandemic, these shelters, heterogeneous in terms of the institutions responsible for them and their orientation, model, degree of care provided, and trajectory also faced complex problems. These included the overpopulation of these spaces, the availability of tangible and intangible resources for their operation, resources often dependent on cross-border solidarity (particularly in the case of shelters run by secular or religious non-governmental institutions, which were the majority), and the fact that these spaces were designed as temporary shelters but had to serve migrants needing long stays and a rotating population such as deportees (8).

On 30 March 2020, Mexico declared a health emergency due to the coronavirus, immediately followed by imposing “stay-at-home” orders that turned temporary migrant shelters into spaces for shelter, voluntary isolation, and quarantine of people on the move along the northern border without a place to “stay home.”

Elements that increase the risk factors for contagion by COVID-19 in shelters include the turnover and overcrowding of people, gathering in communal areas, the need for daily supplies, water service availability, and sanitation. In addition, since April 2020, states on the northern border, mainly Baja California and Chihuahua, having the main border cities, stand out nationwide by their high rates of contagion and deaths from COVID-19. During the 1st weeks of the health emergency, migrant shelters in this region were associated as possible sources of infection, outbreaks, and contexts where prevention, control, and surveillance of the epidemic had become unmanageable. Was this association verified in the early months of the pandemic?

At the end of March and beginning of April 2020, the first response to reduce the spread of infections in these shelters was to “decongest” shelters and go into lockdown, in other words, to close their doors to new migrants, volunteers, and organizations. Some cities set up sanitary filter shelters. On 11 May 2020, the Ministry of Health (SESA) published the *Operating Plan for the Care of the Migrant Population during COVID-19*, in an environment of public health policies focused on coping with the pandemic, at least from January to September 2020 failed to consider the needs of this population (9).

This Plan prioritized the role of the SESA and the Health Jurisdictions, centralized coordination of “comprehensive care” including medical care (pre-hospital, primary, and secondary care); mental health; epidemiological surveillance and laboratories; health promotion; reproductive health and protection) for migrants in conjunction with various agencies in the health sector, the INM (National Migration Institute), NGOs (Non-Governmental Organizations)/ACs (Civil Associations), and local governments, including migrant houses and shelters. Given the common problems faced by migrant houses and shelters pose the following questions: what were the regional challenges of implementing epidemiological surveillance and control guidelines as described in the Plan? How was the follow-up of COVID-19 cases detected in these shelters conducted? Was contagion successfully contained? Using a qualitative approach, we sought to measure the incidence and spread of contagion in the empirical case of migrant shelters in this region. We answered the question above using qualitative research with an ethnographic approach that included conducting 48 semi-structured in-depth interviews with shelter staff and key informants between July and December 2020, in the cities of Tijuana (Baja California), Nogales (Sonora), Ciudad Juárez (Chihuahua), Piedras Negras (Coahuila), and Heroica Matamoros (Tamaulipas).

The main finding is that the shelters did not become sources of contagion, we found only seven out of 78 shelters had confirmed COVID cases, detected through PCR, and only two registered outbreaks. Nonetheless, certain factors may have influenced the epidemiological surveillance process and resulted in underestimating positive COVID-19 cases at these locations.

## 2. Materials and methods

This article presents some of the findings of a research project titled “United States-Mexico Border Health Conditions” financed by the US-Mexico Border Health Commission and El Colegio de la Frontera Norte in Tijuana. The overall objective was to analyze the response to the spread of COVID-19 virus infections from a public health perspective among migrant shelters in cities on the northern border of Mexico. The research was based on a qualitative methodological design using an ethnographic approach. Data collection took place between July and December 2020.

### 2.1. Study areas

The cities on the Mexican northern border chosen to be included in the research met the following qualitative criteria.

- A border city from each Mexican state that has a border with the United States, ~ 3,000 km long, in order to record the particularities of the states' socio-political context. Efforts were made to select the most populous city and the main border crossing point for each state.
- Cities that are immersed in the main mass migratory flows of people tend to use migrant shelters. Includes cities that serve as a port of return for deported migrants and/or asylum seekers in the United States under the MPP (Migrant Protection Protocols), and/or with the largest number of people on the waiting lists for requesting asylum in the U.S., and/or most involved in the transit of undocumented migrants seeking to cross the U.S. border.
- Key cities that bear a presence of a shelter network that provides accommodation services for migrants. We prioritized cities where the network is comprised of shelters with different trajectories (established, recent, emerging) under the aegis of institutions (secular and religious civil society organizations, governments, and international organizations), to record the heterogeneity characterized in the shelter landscape and its patterns along Mexico's northern border. We included major cities having health filter shelters set up for epidemiological surveillance and prevention during the pandemic.

The eligibility criteria resulted in the following cities being included in this study: Tijuana (Baja California), Nogales (Sonora), Ciudad Juárez (Chihuahua), Piedras Negras (Coahuila), and Heroica Matamoros (Tamaulipas).

### 2.2. Study subjects and key informants

The study subjects in this research took refuge in shelters, asylums, foster homes, and, in general, institutions with or without a civil society charter that granted humanitarian support by providing accommodations to a population fully or partly composed of migrants with varying profiles along the five cities on Mexico's northern border.

The key informants were institutional actors who, within the framework of the pandemic, intervened in migrant shelters, provided health care in these spaces, and/or participated in the development of strategies to mitigate infection and follow-up on suspected or confirmed COVID- 19 cases detected in shelters.

Key informants also included parties holding a holistic view of the study focus who were able to describe the practices, patterns, needs, challenges, facilities, and resources related to the response of shelters to the pandemic. Key informants were mainly identified during the research process, through the narratives and networks of the study subjects consulted.

Migrants housed in the northern border shelters were not interviewed for two main reasons. First, the impossibility of physically going to these spaces complicated contact with this population. Second, when the field data collection phase was conducted between July and December 2020, a time when migrants in shelters saw their migratory and life projects negatively affected by the adoption of public health policies designed to stop the spread of contagion in Mexico and the United States.

### 2.3. The study population

As a first step, a list was drawn up with the contacts of active shelters for migrants in the cities under study. These shelters were mapped based on previous academic research that had identified these spaces in the region; the websites and/or social networks of migrant shelters in the cities under study; the lists of institutions that provide humanitarian assistance along the migratory routes drawn up by organizations that support populations on the move. The list was also enhanced by information gathered through interviews with study subjects and key actors.

### 2.4. Research instruments

The research instrument was a semi-structured, in-depth interview conducted in 40 cases by telephone, in five cases through virtual platforms, and in three cases with face-to-face interviews. Out of a total of 48 interviews, 19 were conducted with study subjects (people in charge, managers, coordinators, shelter directors) from 22 migrant shelters, and 30 with key informants. A brief questionnaire was also administered in 74 of the 78 shelters to survey each institution's profile and ensure that it was operational during the time of the research.

Shelter classification was based on the methodology by Albicker and Velazco (10) which categorizes shelters as “pioneering,” “consolidated” and “recent” to describe the range of shelters in Tijuana, which coincided with the influx of Haitian migrants in 2016−2017, recording an increase in shelters in the other cities under study. The founding year is when the shelter began to receive migrants. In all the cities in the study, “pioneering” migrant shelters refer to those having over 20 years of experience and were established in 2000 or earlier. “Consolidated” shelters refer to those that were created between 2001 and 2015 and continue in operation. “Recent” shelters refer to those established from 2016 to the present that were set up to meet recent extraordinary migrant flows mainly from those seeking asylum to the United States which varies by city. For example, in Tijuana, the influx of Haitians in 2016-2017 drove the creation of recent shelters, whereas, in Piedras Negras, it was following the arrival of the migrant caravan in February 2019. Similarly in Ciudad Juárez, a “wave” of Cuban and Central American migrants in late 2018 and early 2019 prompted the creation of recent shelters. In Matamoros and Nogales, the implementation of the MPP at the beginning of 2019 triggered an increase in entrapped migrants in need of long-term accommodation which led to the establishment of new shelters, in these two cities, “recent” shelters are those established in 2019 to the present.

The interview guide for study subjects was divided into thematic axes with 57 guiding questions, broken down as follows: the interviewee profile, shelter profile, migratory context where the shelter operates, participation in institutional networks, and efforts to coordinate health care in the shelter and respond to the pandemic. Other axes included the shelter's first response to the pandemic, prevention measures, epidemiological control, surveillance undertaken, monitoring of suspected cases, confirmed cases, contacts detected, outlook, and intervention proposals.

The interview guide for key actors included thirty-five guiding questions divided into the following thematic axes: interviewee profile, institution profile, the migratory context where the institution operates, information on the reaction of migrant shelters to the pandemic, interventions by the institution to support shelters in the context of the pandemic, inter-institutional coordination, and outreach for the health care of the migrant population during COVID-19, prospects and intervention proposals.

### 2.5. Systematization and analysis

The information gathered was transcribed and systematized into two tables or analytical templates, one for interviews with study subjects and another for interviews with key actors. The information obtained was organized in the tables into several homologous analytical categories. The findings presented in this document are mainly drawn from an analysis of the categories of “epidemiological monitoring of suspected COVID-19 cases detected in shelters” and “epidemiological surveillance and prevention in shelters.” These categories, contained in both templates, were used to analyze the material gathered through the other instruments.

## 3. Findings

### 3.1. Shelters on the northern border of Mexico during the lead-up to the pandemic: Heterogeneity and common problems

The following findings emerged:

- Increase in emergency shelters to meet the demand for accommodation of the recent large, extraordinary flows, especially of people seeking international protection, refer to Table 1.
- Heterogeneity of institutions in charge, many types of institutions, and heterogeneity within the same type (international organizations, governments, secular non-governmental institutions, both Protestant (Baptist, Methodist, and Pentecostal) and Catholic (Jesuit, Salesian, and Scalabrinian).
- Different models and degrees of care. Some shelters offered basic care (accommodation, toilets, food, and clothing/shoes), and other shelters offered expansive services (such as accommodation, food, clothing, medical care, education, legal advice, and employment services). These shelters provided comprehensive care to integrate the migrant population into the city (such as Tijuana and Ciudad Juárez's integration centers and certain Migrant Houses).
- Assorted sizes and maximum capacities: small (family) shelters with fewer than 50 people, such as El Puente in Tijuana, and massive shelters for nearly 500 people such as San Juan Bosco in Nogales, or 1,000 or more such as Pan de Vida in Ciudad Juárez and those repurposed by the government on the premises of former maquiladoras.
- Shelters' adaptation to new user profiles due to the increase of displaced unaccompanied Children and Adolescents (CA), single women and women with children, and families with CA.
- The pandemic reinforced the tendency to eliminate the maximum length of stay in the regulations and the maximum time was adapted to the migratory process for each person for whom accommodation was provided.
- Implementation of confinement policy in shelters that became a “co-responsible domestic shelter” space for people on the move, that is, those without a fixed address in the northern Mexican border region.
- Heavy dependence on shelters run by non-governmental institutions offering cross-border solidarity.
- Epidemiological filter shelters were set up as a result of the pandemic. In both the city of Tijuana and Ciudad Juárez, the International Organization for Migration (IOM) Filter Hotel was adopted. In Ciudad Juárez, two filter spaces operated by a religious non-profit (the San Matías Shelter System and the Espíritu Santo Shelter) were adopted.

**Table 1:** Pioneering, consolidated, and recent shelters in the cities under study July–December 2020.

**City**	**Pioneering**	**Consolidated**	**Recent**	**Total**	**%**
Piedras Negras	3	4	1	8	10.2
Nogales	1	2	2	5	6.4
Matamoros	3	0	3	6	7.7
Ciudad Juárez	5	4	13	22	28.2
Tijuana	11	12	14	37	47.4
Total	23	22	33	78	100
%	29.5	28.2	42.3	100	

Source: Compiled by the authors based on the information collected and according to the proposal put forward by Albicker and Velasco (10).

### 3.2. Managing the pandemic in shelters

#### 3.2.1. COVID-19 shelter guidelines

During the early weeks of the health emergency, the epidemiological containment and prevention measures adopted in the shelters resulted from informal consultations and inquiries between responsible parties and local health authorities. As of 19–20 March 2020, federal level and Health Jurisdictions required shelters to implement quarantine measures, the refusal of entry to new migrants, volunteers, and members of organizations supporting these institutions, and the “decongestion” of spaces by relocating residents. This was the general trend in the initial response to the pandemic by shelters located on the northern border of Mexico.

In response to the pandemic, from January to September 2020, public policies and government health initiatives largely excluded the migrant population from health care (9). On 11 May the Ministry of Health published the *Operating Plan for the Care of the Migrant Population during COVID-19* whose main purpose was to “establish effective coordination and liaison for comprehensive health care for the migrant population during COVID-19” (11), particularly in the northern and southern border regions of the country where the target population is concentrated.

This plan prioritized the role of SESA and the Health Jurisdictions, which were charged with coordinating “comprehensive care” for this population, which would be guaranteed in conjunction with the various agencies in the health sector, the INM, NGOs, and state and municipal governments. Migrant houses and shelters, together with points of entry into Mexico and health sector units, were included in the areas of action of the Plan.

Health jurisdiction brigades identified migrant shelters and visited them to establish links, register their population, provide epidemiological guidance, and disseminate information on COVID-19 (through posters and brochures) and the detection of symptoms. They offered guidelines on how to clean up shelters and adopt prevention measures (suggesting ways to adapt the infrastructure to implement physical distancing measures) and provided supplies for prevention and personal protection.

When a case with symptoms related to SARS-CoV-2 virus infection was detected in a shelter in the cities under study, the protocol described in the plan called to immediately notify the city's health jurisdictions for each individual case. The jurisdictions would be responsible for implementing actions and mechanisms to verify the event, surveillance, and laboratory, and ensure the care and follow-up of suspected and confirmed cases and contacts based on their surveillance, control, and epidemiological prevention criteria.

However, the standardized protocol proposed by SESA merely “provided guidelines” for actions and decisions for shelter administrators and personnel in the Health Jurisdictions. In reality, the assigning of the follow-up process by the actors involved led to different, circumstantial, specific care routes, and follow-up practices for each suspected case detected in the shelters in each city.

The factors discussed above led to high uncertainty and improvisation around actions implemented with each circumstance. This was exacerbated during the 1st months of the pandemic when the official guidelines were barely disseminated, which in turn affected compliance with control criteria and epidemiological surveillance.

#### 3.2.2. Early detection and assessment of cases at shelters

The preventive actions recommended by the *Operating Plan for the Care of the Migrant Population* (11) for migrant shelters included a health supervision filter involving the implementation “in all cases” of triage, and a questionnaire to detect signs and symptoms. Respiratory triage was presented as an instrument designed to detect suspected COVID-19 cases and determine the urgency of care.

Furthermore, “triage” means that the institutions implemented the questionnaire and administration model provided by the Ministry of Health systematically and/or for each new admission and were limited to the following:

—Shelters with specialized medical staff responsible for primary care, such as Migrant Houses and the IOM Filter-Hotels.

—Government shelters where permanent medical care was dispensed by the health sector, such as the Integration Centers for Migrants where IMSS, ISSSTE, and SESA doctors provide service.

—Shelters where the Jurisdiction emphasized the training and hiring of health promoters trained to detect symptoms and implement the immediate follow-up phase, as in the case of the Espiritu Santo and San Matías filter shelters in Ciudad Juárez.

Very few of the shelters interviewed were aware of, or administered this instrument. However, they all set up a sanitary filter at the entrance including a registration questionnaire, supplying antibacterial gel, and in some cases, taking temperature and blood oxygen measurements.

However, the fact that the “triage” stipulated by the Health Ministry was not administered or known in the shelters did not stop them from developing instruments and mechanisms for detection and epidemiological control during the pandemic. For example, in the written or oral registration questionnaire where several shelters recorded general information on the migrant and their migratory trajectory at the time of admission, some institutions increased the number of questions regarding their health status. These included questions on chronic degenerative pathologies, living with positive or suspected COVID-19 people, and the presence of the main symptoms of COVID-19.

Shelters that notified authorities of cases reported that the assessment of the event by the Jurisdiction had taken place in an isolated space in the shelter, or another designated area (such as the Fever Clinics in Tijuana or the Centinela Anticipa Unit Clinics in Nogales), and included the administration of a combination of instruments, including the “respiratory triage,” and the search for cases with fever. If a person fits the operational definition of a suspected case, an epidemiological study of a suspected case of viral respiratory disease was conducted, and contact tracing began. In addition, assessment could include the collection of a sample for the administration of a rapid test and/or the collection of a sample for a PCR laboratory test. Appropriate isolation measures were subsequently determined. In all the cities under study, the health authorities' evaluation policy regarding a case with symptoms at a migrant shelter only focused on the administration of a diagnostic test in limited cases.

The PCR, the prerogative of the Jurisdictions, tended to be administered to just a small fraction of suspected cases with obvious respiratory symptoms. This may have led to the underreporting of infections at migrant shelters in the official statistics of the Jurisdictions, which are based on positive PCR results.

#### 3.2.3. The isolation of migrants with “suspected” and “confirmed” COVID-19 detected in shelters

One problem from the start of the pandemic and creating enormous concern among responsible parties for the shelters was the need to have an isolated or quarantine area for suspected and confirmed COVID-19 cases, those with mild symptoms, and contacts detected among the migrant population housed in these shelters.

Isolation is required from the time between the detection of a case with symptoms and the confirmation of the event by health authorities at the shelter. When a PCR test was administered, the person remained isolated until the laboratory results were received (from 2 to 4 days depending on the distance from the laboratory). Once the laboratory confirmed a person had tested positive or had been in contact with someone who had (confirmed by epidemiological association), isolation was extended for 2 weeks. This measure was applied to all suspected cases, even when they had not been given a rapid test or laboratory diagnosis.

At the beginning of the pandemic, very few shelters in the study had a designated isolation area meeting necessary requirements such as having separate bathrooms and sufficient space to ensure that “asymptomatic” and “confirmed” cases would not be lumped together with “contacts” (11).

Having a space for isolation depended on a combination of factors such as the availability of space, and the human and financial resources to ensure isolation and provide medical/clinical follow-up to those who needed it, in addition to the resources for the total daily support of the person in quarantine for at least 2 weeks.

The lack of this space, coupled with the impossibility of supporting the process of monitoring COVID-19 cases, forced many shelters to make the decision to close or continue operating behind closed doors to minimize the possibility of becoming a source of infection during the pandemic.

To continue operating and accepting new migrants, some shelters, such as the Casa del Deportado de Tijuana and La Roca de Nogales shelter turned one of their dormitories into an isolation space that could be used in an emergency.

Shelters equipped with a space for the isolation of suspected and confirmed asymptomatic cases, or those with mild symptoms—which do not require secondary care (confirmed cases with severe symptoms are referred to the isolation units of General Hospitals or other COVID-19 units set up in each city) —were characterized by the following:

—Certain Migrant Shelters had areas for isolation with a large backyards.

—Shelters supported by international organizations and/or the government have an isolation area for all suspected cases: “it is a small space. Only the doctors who are there go there all the time with all the necessary equipment and take in food, and from the time they are suspected cases to the time when they take the test, they are not allowed to leave” (telephone interview with a shelter's responsible party, 25 September 2020).

—Other shelters with large areas were set up.

Most organizations found it impossible to deal with this problem autonomously and immediately. In the 1st weeks of the health contingency, the OIM activated the Program to Strengthen Shelters (The Shelter Program was valid for 22 months ending in April 2021) (12) to meet the need, lending canopies to the shelters that required them to isolate people with symptoms during the evaluation phase or confirmed cases indefinitely.

Although some shelters did not fully resolve the need for confinement and containment of contagion given events of a severe outbreak in the establishment, as was mentioned by some of the responsible parties we consulted, the IOM canopies managed to allay widespread fear and uncertainty about how to proceed, providing a solution for the initial isolation of confirmed and suspected cases detected in shelters.

In Tijuana and Ciudad Juárez, the creation of filter shelters such as the IOM Filter-Hotels or the San Matías and the Espiritu Santo prevented an outbreak of COVID-19 cases in the shelter network. Moreover, in each city under study, care referral routes were established for each case with symptoms detected in the shelters, which included their immediate transfer to other establishments equipped with specific isolation spaces, in some cases from the time of the assessment phase. In each city, these spaces consisted of several types of shelters not necessarily exclusively intended for the migrant population, or the shelter population, such as Voluntary Isolation Centers (CAV), mobile clinics, COVID-19 Units at General Hospitals, and COVID-19 Centers set up by the Ministry of Health, often in collaboration with other actors such as Doctors Without Borders and the private sector.

Exercising epidemiological control through isolation posed a challenge for most shelters, which generally had limited, unreliable support, due to the demand for efforts and resources required for monitoring, even if they had a suitable space for doing so. But it was also a challenge for those housed at the shelter.

For migrants, isolation meant separation from friends and family, with the displacement of the entire family nuclei. It meant being away from places with a flow of vital information. As noted by a doctor from the Ciudad Juárez jurisdiction: “often if people at the shelters […] have an appointment with the MPP, they do not want to be isolated, because what if they call them about the MPP when they are in isolation?”

In addition, the need for quarantine and isolation created pressure on the shelters that housed migrants, especially in the areas controlled by the government or with a larger population, such as the Ciudad Juárez Migratory Integration Center and the Matamoros Camp, which housed ~2,500 migrants at the start of the pandemic, most of them families seeking asylum in the United States. In some cases, the pressure led to protests, sit-ins, riots, plots, and uprisings, as noted by key respondents.

In Nogales, eleven migrants were isolated in an area of the DIF (National System for Integral Family Development) Municipality that had resulted from a contagion outbreak in a shelter that continued to operate as one of the few accommodation options available to migrants in the city. The key actors interviewed reported difficulty in maintaining and sustaining isolation for 2 weeks due to the lack of government budget funds specifically designated to serve this population. The cost of a portion of the supplies was partly covered by the personal salaries of municipal officials and support from a local civil society organization “Panchito y Su Cristina,” supported by the American NGO, Voices from the Border. In addition, it was not possible to have personnel permanently monitor the area or confinement of migrants nor to separate lab-confirmed cases and their contacts. Nonetheless, in all of the cities, the isolation measure was applied to all suspected cases that were detected at the shelters, even when they had not been administered a rapid test or had a laboratory diagnosis.

During the early months of the pandemic, the implementation of the protocol for epidemiological monitoring of migrants with suspected and confirmed COVID-19 acquired a connotation of uncertainty and improvisation that negatively influenced the way the criteria for surveillance, prevention, and control of the epidemic were implemented.

### 3.3. Contagion at the shelters in numbers: Underestimation in official statistics

#### 3.3.1. Tracking the tests administered

We found heterogeneity in the data figures from the actor interviews in shelters in the cities under study used to estimate the degree of infection. The issue of epidemiological surveillance of the virus among the shelter population was also complicated by the lack of a reliable official record.

The health authorities' event evaluation policy applied in the cities under study included the administration of a PCR (Polymerase Chain Reaction) test for a few cases in migrants showing obvious or severe respiratory symptoms. According to estimates by the Health Jurisdictions, by December 2020, PCR tests confirmed 40 cases recorded at the shelters, an estimate matching the figures shared by the jurisdictions' staff. The estimate above excludes the city of Tijuana because their jurisdiction did not share its figures. The Directorate of Care for Migrants for the Municipality of Tijuana reported two cases were confirmed by PCR tests in the shelters.

In addition, during the 1st months of the pandemic shelters had limited access to rapid tests and there were no official records of rapid tests administered in migrant shelters in the cities studied. Thus, tracing the route of PCR tests or rapid tests that were applied did not yield a reliable record of infections in the shelters and both routes seemed to lead to an underestimate of infections.

In regards to the scope of the contagion in Tijuana and Matamoros, there was not a marked difference in the figures provided by the actors responsible for receiving migrants. In Piedras Negras, no cases were registered in these spaces by any actor interviewed since the shelters stopped working under the city council's order. In Ciudad Juárez, where the state tends to centralize the coordination of the humanitarian sector and strengthen the link between shelters and the Health Jurisdiction, figures provided by the actors interviewed tended to be similar to official figures.

The Health Jurisdiction estimates considered cases that had tested positive with the PCR test. The numbers declared by other actors came from information based on rapid tests administered directly or indirectly by them and could include cases that tested positive with the rapid test, and/or isolated cases, and/or cases identified as positive as a result of an observation of symptoms assessment.

The difficulty of having reliable information on the spread of infection in shelters could also be due to political factors, and the inaccurate, non-transparent handling of data between different actors, levels, and areas of government.

Reflecting on the following account by a doctor from the Health Jurisdiction (face-to-face interview on 26 October 2020), one can assume that in Tijuana, transferring migrants with suspected COVID-19 between various COVID-19 care centers may also have complicated the estimates:

“There were positive cases, but there were no serious cases we had to hospitalize. They were just patients we had to transfer at the time we had to refer them to the General Hospital. Once they had recovered at Zonkeys (Zonkeys basketball stadium, in Tijuana, where an auxiliary hospital was set up to care for patients with COVID-19) Hospital or the COVID-19 Shelter, they were usually transferred from the General Hospital to Zonkeys and from Zonkeys to the COVID-19 shelters; there was only one case of hospitalization.” Thus, contrary to speculation during the first weeks of the pandemic, migrant shelters on the northern border did not become sources of COVID-19 infection, since from the seventy-eight shelters analyzed, only seven had confirmed cases, and only two received the classification of “outbreaks”.

Infections were recorded tendentially at the shelters housing most migrants, (such as the San Juan Bosco de Nogales or Campamento de Matamoros) where monitoring was undertaken by staff doctors or specialized medical personnel from the government health sector (such as the CIM in Ciudad Juárez and the Filter Hotels—OIM). Based on the interviews, the people who were isolated came from groups of returnees from the United States and asylum seekers under the MPP. Only two cases of isolated migrants in the cities studied were referred to General Hospitals due to major complications, and both successfully recovered. In most cases where migrants had been isolated were eventually reincorporated into the community and their shelter after a fortnight, when they did not present symptoms after testing negative, but they did not always administer a confirmatory PCR test.

#### 3.3.2. Circumstantiality of care routes and the threat of quarantine for shelters

Another factor that may have contributed to the underestimation of the official data on people infected by COVID-19 in shelters is due to certain institutions using a different protocol, follow-up, or care route than that established in the guidelines for suspected cases.

Key informants reported that shelters with internal medical personnel and isolation spaces preferred to treat and manage cases with mild symptoms discreetly, isolating them in their facilities without sharing information with health authorities. At the same time, Civil Society Organizations that provided health care in the shelters declared that they were the first and only contact in the event of suspected cases at certain institutions and preferred not to interact with health authorities. The director at one of these organizations commented: “ I know the Jurisdiction is trying to do its job, but the simple fact that they wear a uniform prevents them from having access to the shelters […] the population doesn't trust them [...] and neither do the shelters, especially those that are illegal or clandestine, and are reluctant to let them in.”

Fear of the imposition of quarantine or other repercussions may have also discouraged institutions from accessing and notifying suspected cases of the Health Jurisdiction. Notification of a suspected case among migrants or workers and volunteers could imply a quarantine for the entire institution, with the obligation to assume responsibility for the entire sheltered population, which posed an enormous challenge for shelters whose survival depended on a combination of limited and uncertain resources.

#### 3.3.3. The implications of isolation for migrants

A final factor that may have led to the underestimation of infections in the shelters is linked to a trend observed among housed migrants to not disclose the onset of COVID-19 symptoms to the shelter or camp staff. Actors who provided services in the Ciudad Juárez CIM and the Matamoros camp observed this attitude and associated it with the fear of being kept in an isolation space, which would exacerbate the loss of control of their projects which were already profoundly disrupted by health and immigration policies adopted in Mexico and the United States during the pandemic.

Moreover, migrants may have been discouraged from reporting symptoms given a perceived lack of clarity and transparency in protocol compliance. The sensitive nature of the protocol for monitoring suspected cases and its implementation created uncertainty about what lay in store for migrants in the event they became a “suspected COVID-19 case.”

## 4. Conclusion and discussion

In the past 5 years, cities on the Mexican northern border have received extraordinary flows of foreign and Mexican migrants in transit to and from the United States, with even more complex and diversified profiles and needs including people from various countries (Honduras, El Salvador, Guatemala, and Haiti), an increase in the presence of children and adolescents, women traveling alone with their children, and more families displaced by violence within Mexico. At the same time, the implementation of security, immigration, and health policies by the U.S. and Mexico transformed this border into the last filter to contain these flows (2, 13–20).

This situation called on the humanitarian system that is active on the northern border of Mexico to provide care for this population on the move, stuck on the move, or in a condition of “forced mobility,” as it has been classified by several analysts (7, 16).

One of the pillars of this humanitarian system is the range of options offering accommodation to migrants who are highly susceptible to the recent sudden, drastic changes in the migratory dynamics of this border region and fluctuations in the demand for housing (21, 22).

Recently, the array of shelters in this region assumed the appearance of a heterogeneous, fragmented body in terms of the type of structures, responsible institutions, and operating models. At the same time, they shared problems that, during the first few months of the pandemic, challenged the control and containment of contagion in these spaces, as well as the application of epidemiological surveillance protocols and case monitoring.

We attempted to measure the incidence and spread of contagion using a qualitative approach in five cities on the northern Mexican border. We also analyzed the phases of the epidemiological monitoring process for suspected COVID-19 cases detected in these spaces which are, detection, assessment of the event, and isolation. In each phase, we highlighted the factors (social, economic, cultural, and political) that influenced the appropriation of epidemiological surveillance protocols in these spaces. We found that the difficulty of having reliable information on the spread of infection in shelters could also be due to political factors and the inaccurate, non-transparent handling of data between different actors, levels, and areas of government.

Contrary to speculation, during the early weeks of the pandemic migrant shelters in the northern border did not become sources of COVID-19 infection, given that in a total of seventy-eight shelters in the five cities studied, only seven showed confirmed cases, and two shelters received the classification of “outbreaks.” Thus, contagion control or containment was successful.

A total of 81% of the 42 cases confirmed through PCR tests given by health authorities were concentrated in Ciudad Juárez. From this figure, 52% were detected in the Integration Centers for Migrants and 33% in the OIM Filter Hotels. These shelters continued to operate and accept new migrants but also had permanent staff and professional medical health care which became a tool to detect infection.

In addition, the implementation of strategies for control and containment materialized such as non-profit shelters operating behind closed doors and accepting new admissions, the implementation of epidemiological filter shelters by religious non-profits and international organizations in Tijuana and Ciudad Juarez, and the adoption of a preventive isolation policy. A preventive containment logic was detected which included the isolation of all suspected, even unconfirmed, cases of COVID-19 among migrants. At the same time, a lack of transparency and clear agreements was observed regarding the human and financial resources required to maintain isolation spaces, which were often improvised (as in the case of Nogales and the Matamoros Camp).

However, the manner in which study contexts appropriated epidemiological surveillance and control protocols incorporated elements that hampered surveillance in these spaces and led to an underestimation of the phenomenon. A comparison of the information provided by the health authorities with that of the shelters and key local non-governmental actors with more contact with the field revealed higher underestimation rates in the cities of Tijuana, Nogales, and Matamoros. The factors that contributed to this underestimation were:

- Circumstantiality of protocols in each city under study during this initial stage of the pandemic. There was a lack of clarity about assistance routes, what happened to the migrant when they became a suspected case, and what happened to the shelter when a suspected case was detected.
- Migrants' and shelters' fear of quarantine and isolation.
- The incipient relationship between the health sector and shelters materialized in the shelters' fear of being sanctioned or “controlled” by health authorities.
- Limited availability of human resources and medical-health personnel in shelters, exacerbated by safe distance and shelter-in-place policies during the pandemic.
- Limited availability and administration of PCR tests when Jurisdictions exceeded their intervention capacity.

The factors mentioned above along with their association with social, political, economic, and administrative spheres reveal the criticality that emerged from observing the planned standardized surveillance protocols in this heterogeneous overview of shelters. During the early months of the pandemic, shelters in the cities studied managed to contain the contagion while serving as spaces to shelter in place, quarantine, and offer access to some form of medical care in the event of contagion given an institutional environment that was closed to the population on the move. However, this study did not record the opinions of migrants at the shelters and it remains a pending task.

### Limitations

The travel restrictions imposed by coronavirus infection prevention and containment measures made it impossible to engage in an on-site stay in the cities in the study, with the exception of Tijuana. As a result of these limitations, this study was an ethnography “at a physical distance” that drew information from telephone interviews, virtual platform interviews with study subjects and key informants, and from a critical hemerographic review of local newspaper articles on the subject. We also accessed documents published by academia, and local and international non-governmental organizations focusing on the issue of care at migrant shelters for the population on the move along Mexico's northern border during the health contingency. Finally, we also reviewed official documents and communication that established care guidelines for these spaces during the pandemic.

## Data availability statement

The datasets generated for this study are available on reasonable request to the corresponding author.

## Ethics statement

The studies involving human participants were reviewed and approved by the Ethics Committee of the United States-Mexico Border Health Commission. Subjects provided their informed consent to participate in this study.

## Author contributions

MR and RC-P: article proposal and review. VC: fieldwork and first and final draft document preparation. AL: document review and editing for Frontiers. MR, RC-P, and AL: final draft preparation. All authors contributed to the article and approved the submitted version.

## References

[1] Organización, Mundial de la Salud (OMS). Actualización de la estrategia frente a la COVID-19. Available online at: https://www.who.int/es/emergencies/diseases/novel-coronavirus-2019/strategies-plans-and-operations (accessed August 13, 2021).

[2] Musset A. De los lugares de espera a los territorios de la espera. Una nueva dimensión de la geografía social? Documents d'Anàlisi Geogràfica. (2015) 61:305–24. Available online at: https://raco.cat/index.php/DocumentsAnalisi/article/view/292867

[3] Jasso V Rosalba. Espacios de estancia prolongada para la población migrante centroamericana en tránsito por México. Frontera Norte. (2021) 33:4. 10.33679/rfn.v1i1.2075

[4] Del Monte A y París Pombo D. Informe sobre las condiciones de estancia en el campamento de refugiados del Chaparral en la frontera de Tijuana. Observatorio de Legislación y Política Migratoria. (2021). Available online at: https://observatoriocolef.org/boletin/informe-sobre-las-condiciones-de-estancia-en-el-campamento-de-refugiados-del-chaparral-en-la-frontera-de-tijuana/ (accessed August 3, 2021).

[5] Unidad de Política Migratoria. Boletín Mensual de Estadísticas Migratorias. México: Secretaría de Gobernación, Gobierno de México (2020).

[6] Odgers-Ortiz O. The perception of violence in narratives of central american migrants at the border between Mexico and the United States. Revue européenne des migrations internationals. (2022) 36. 10.4000/remi.14452

[7] Coubès ML Velasco L Contreras OF. Migrantes en albergues en las ciudades fronterizas del norte de México. In: Contreras OF, ed. Ciencias Sociales en acción: Respuestas frente al Covid 19 desde el norte de México. Mexico: El Colegio de la Frontera Norte. (2020) 2020:340–57.

[8] Fundación para la Justicia y el Estado Democrático de Derecho. Informe sobre los efectos de la pandemia de COVID-19 en las personas migrantes y refugiadas violaciones a derechos humanos documentadas por organizaciones defensoras y albergues en México. México: CDMX (2020).

[9] Bojorquez-Chapela I Strathdee SA Garfein RS. The impact of the COVID-19 pandemic among migrants in shelters in Tijuana, Baja California, Mexico. BMJ Global Health. (2022) 7:e007202. 10.1136/bmjgh-2021-007202 35277428 PMC8919131

[10] Albicker S Velasco L. Capacidades de la sociedad civil en Tijuana para atender y proteger a la población migrante. In París Pombo MD, Migrantes haitianos y centroamericanos en Tijuana, Baja California, 2016–2017. Políticas gubernamentales y acciones de la sociedad civil. Tijuana: Comisión Nacional de los Derechos Humanos/ El Colegio de la Frontera Norte (2019) (pp. 53–65).

[11] Secretaría de Salud. Recomendaciones sanitarias para refugios temporales y Centros de Aislamiento Voluntario (CAV) en el contexto de Covid-19. México: Subsecretaría de Prevención y Promoción de Salud (2020).

[12] Agencia de las Naciones Unidas para los Refugiados.Instala ACNUR unidades de vivienda en albergues y centros de salud en norte y sur de México. (2020). Available online at: https://www.acnur.org/noticias/press/2020/7/5f1f20d44/instala-acnur-unidades-de-vivienda-en-albergues-y-centros-de-salud-en-norte.html (accessed January 3, 2020).

[13] París Pombo D. El cierre de la frontera estadunidense y los solicitantes de asilo bloqueados en el norte de México. Nexus. (2020). Available online at: https://migracion.nexos.com.mx/2020/10/el-cierre-de-la-frontera-estadunidense-y-los-solicitantes-de-asilo-bloqueados-en-el-norte-de-mexico/> (accessed February 25, 2021).

[14] Pombo MD Carnero EI. La externalización del asilo a la frontera Norte de México: protocolos de protección al migrante. En Migraciones en México: fronteras, omisiones y transgresiones Informe 2019. Ciudad de México: REDODEM (2021).

[15] Núñez G Heyman J. Comunidades de inmigrantes “atrapadas” en los procesos de control de la libre circulación: consecuencias de la intensificación de la vigilancia en la zona fronteriza. In Natalia Armijo Canto (Ed.), Migración y Seguridad: nuevo desafío en México. México: Colectivo de Análisis de la Seguridad con Democracia (2007).

[16] Odgers-Ortiz O Campos-Delgado A-E. Figeés dans le mouvement : périodes et espaces d'attente des migrants mexicains expulsés des États-Unis. Revue européenne des migrations internationals. (2014) 30:113–35. 10.4000/remi.6922

[17] Ruiz A. Un año después del Acuerdo Estados Unidos-México: La transformación de las políticas migratorias mexicanas. (2020). Available online at: https://www.migrationpolicy.org/research/un-ano-acuerdo-estados-unidos-mexico (accessed August 3, 2021).

[18] Armenta A. Protect, Serve, and Deport. The Rise of Policing as Immigration Enforcement. Oakland: University of California Press (2017).

[19] Department of Homeland Security (DHS). Migrant Protection Protocols. (2019). Available online at: https://www.dhs.gov/news/2019/01/24/migrant-protection-protocols (accessed March 12, 2021).

[20] Ariel R. Un año después del Acuerdo Estados Unidos-México: La transformación de las políticas migratorias mexicanas. (2020). Available online at: https://wwwmigrationpolicyorg/research/un-ano-acuerdo-estados-unidos-mexico (accessed August 3, 2021).

[21] Comisión Nacional de los Derechos Humanos. Los desafíos de la migración y los albergues como oasis. Encuesta Nacional de personas migrantes en tránsito por México. Mexico: CNDH-México, IIJ-UNAM, UNAM (2018).

[22] Rincón Gaburdel E. La sociedad civil organizada responde al impacto de políticas públicas: Las políticas sociales como factor causal del rol asistencialista de organizaciones de la sociedad civil (OSC) pro migrantes en Tijuana. Gestión y política pública. (2018) 27:181–209. Available online at: http://www.scielo.org.mx/scielo.php?script=sci_arttext&pid=S1405-10792018000100181&lng=es&tlng=es

